# Metabolomic Insight into Polycystic Ovary Syndrome—An Overview

**DOI:** 10.3390/ijms21144853

**Published:** 2020-07-09

**Authors:** Anna Rajska, Magdalena Buszewska-Forajta, Dominik Rachoń, Michał Jan Markuszewski

**Affiliations:** 1 Department of Biopharmaceutics and Pharmacodynamics, Medical University of Gdańsk, Hallera 107, 80-416 Gdańsk, Poland; anna.rajska@gumed.edu.pl (A.R.); m.buszewska-forajta@gumed.edu.pl (M.B.-F.); 2 Department of Clinical and Experimental Endocrinology, Medical University of Gdańsk, Dębinki 7, 80-211 Gdańsk, Poland; dominik.rachon@gumed.edu.pl

**Keywords:** metabolomics, polycystic ovary syndrome (PCOS), metabolites, biomarkers, mass spectrometry

## Abstract

Searching for the mechanisms of the polycystic ovary syndrome (PCOS) pathophysiology has become a crucial aspect of research performed in the last decades. However, the pathogenesis of this complex and heterogeneous endocrinopathy remains unknown. Thus, there is a need to investigate the metabolic pathways, which could be involved in the pathophysiology of PCOS and to find the metabolic markers of this disorder. The application of metabolomics gives a promising insight into the research on PCOS. It is a valuable and rapidly expanding tool, enabling the discovery of novel metabolites, which may be the potential biomarkers of several metabolic and endocrine disorders. The utilization of this approach could also improve the process of diagnosis and therefore, make treatment more effective. This review article aims to summarize actual and meaningful metabolomic studies in PCOS and point to the potential biomarkers detected in serum, urine, and follicular fluid of the affected women.

## 1. Introduction

### 1.1. Polycystic Ovary Syndrome

**Figure 1:**
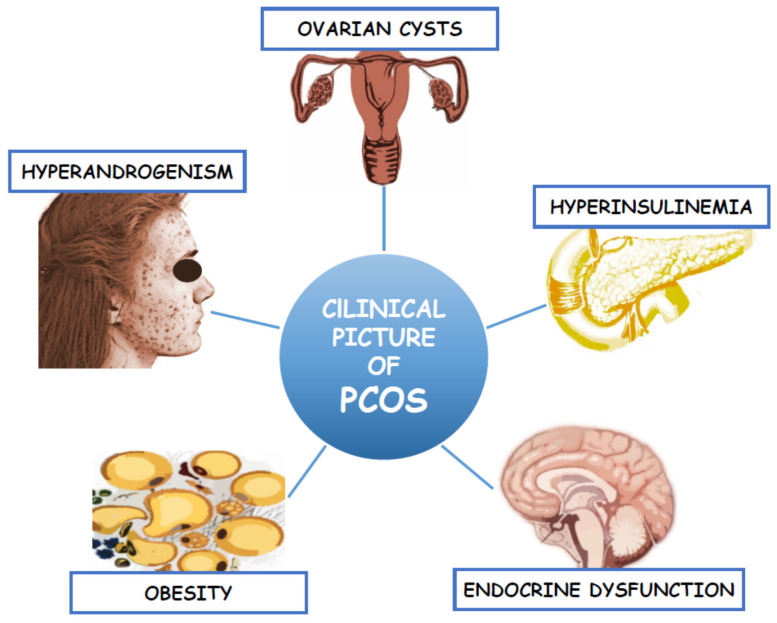
Clinical picture of Polycystic Ovary Syndrome.

Polycystic ovary syndrome (PCOS) is a complex endocrinopathy, which affects more than 10% of women of reproductive age [1]. It is the main cause of female infertility due to oligo- or anovulation. Despite such a high incidence, the pathogenesis of PCOS is still unexplained. Some studies suggest that it is due to the genetic factors associated with ovarian steroidogenesis [2]. According to the Androgen Excess and PCOS Society (AE&PCOS), the diagnosis of PCOS should be based on the presence of clinical and/or biochemical hyperandrogenism (HA) and the ovarian dysfunction defined as menstrual abnormalities (anovulatory oligomenorrhea (AnO)) or/and the presence of the polycystic ovary morphology (PCOM) in the transvaginal ultrasound (TV-US) [3]. These criteria yield three separate PCOS phenotypes: A, B, and C. Phenotype A includes all the three features (HA, AnO, and PCOM) whereas phenotype B and C only two (HA and AnO or HA and PCOM, respectively). However, regarding Rotterdam criteria, the fourth phenotype (D) was separated to comprise AnO and PCOM presence. The clinical symptoms of hyperandrogenism include hirsutism (present in 60% of women), androgenic alopecia, and acne, which negatively affect women’s psyche, their femininity and lead to low self-esteem and depression [4]. In addition to the reproductive and endocrine dysfunction, PCOS is characterized by intrinsic insulin resistance (IR), which lead to the development of the metabolic syndrome (MetS) and its consequences such as disturbed carbohydrate metabolism and type 2 diabetes mellitus (T2DM). Most common clinical manifestation in PCOS is abdominal obesity, which is involved in the development of dyslipidemia, arterial hypertension (AH), as well as non-alcoholic fatty liver disease (NAFLD) [5,6,7,8]. These in turn lead to the development of cardiovascular disease (CVD), which still remains the main cause of death among women [9]. The clinical picture of this complex endocrinopathy was presented in Figure 1. Therefore, the treatment of PCOS focuses not only on the symptoms of hyperandrogenism and infertility, but also on improving IR and its metabolic consequences [10]. Thus, there is a need for a better understanding of the pathomechanisms of this complex disorder through the identification of potential biomarkers with the use of new, non-invasive and specific methods. In recent years, one of the developing scientific approaches is metabolomics [11].

### 1.2. Metabolomic Approach in Studying the Pathogenesis of Polycystic Ovary Syndrome

Among “omics” techniques, metabolomics plays an important role in studying the potential mechanisms responsible for the development of PCOS. Metabolomics allows to identify and quantify small molecules, which occur in all living organisms [12]. The set of all human metabolites that have been identified so far is stored in the Human Metabolome Database (HMDB). Each year, the number of identified metabolites grows. Few years ago, about 41,000 metabolites were found, but now this database contains over 114,190 compounds. Among them, the following groups can be found: amino acids, lipids, peptides, vitamins, organic acids and both endo- and exogenous carbohydrates. Therefore, metabolomics serves as a valuable source of information. The metabolome indicates not only a genetically determined phenotype, but also points to the differences determined by other factors, such as age, diet, or physical activity. The application of metabolomics enables monitoring of the state of an organism and provides information on the compounds formed as a result of many biochemical processes. Any disturbances occurring in a living organism cause changes to the qualitative and quantitative profile of the metabolites. The metabolome describes both the physiological and pathological state of the organism. For this reason, it is known to be an attractive approach, compared to genomics and proteomics, which only suggest the presence of metabolic derangements that occur in the organism [13,14,15,16]. Due to this fact, the use of metabolomics in studying the pathophysiology of PCOS allows to monitor even the smallest biochemical changes in this endocrinopathy and therefore, may help in its diagnosis [17].

Among the many analytical techniques, chromatography coupled with mass spectrometry (MS) seems to be the “gold technique”. While chromatography allows for the separation of metabolites present in complex, biological samples, mass spectrometry provides specific information about the chemical structure of the compounds, such as characteristic fragmentation ions, accurate mass, and isotope distribution pattern utilized for the identification of metabolites. MS characterizes very high selectivity and sensitivity that allows to detect and measure trace amounts of metabolites [18]. The combination of MS with gas chromatography (GC-MS) and liquid chromatography (LC-MS) enables to analyse complex biological samples broadly used in metabolomics. GC-MS is suitable for volatile and non-volatile compounds, which require a derivatization step, but first of all thermally stable analytes. The LC-MS technique is widely used for targeted and non-targeted metabolomic analysis and allows to qualify and identify more polar compounds [19]. Nuclear magnetic resonance (NMR), despite its lower sensitivity than MS, allows to analyse metabolites that are difficult to ionize or require derivative reaction for MS and identify compounds with the same masses [20]. A combination of these complementary techniques enables to analyse a broader array of metabolites and offers more certain results than their separate use.

### 1.3. Matrices for Metabolomic Studies

The application of metabolomics allows the use of several matrices such as tissue and body fluids (i.e., plasma, serum, saliva, follicular fluid, semen). The choice of the matrices is associated with the aim of the conducted study as well as the characteristics of the studied disorder. Ovarian tissue can also be used; however, sampling is invasive and problematic. It is usually obtained during laparoscopic wedge resection surgery. For this reason, the use of ovarian tissue in studying the pathophysiology of PCOS is not very common. The matrices widely used in metabolomic studies associated with PCOS are plasma and urine. Serum and urine samples are more common, because they are easily collectible and simple to prepare. On comparing the significantly altered metabolites, it can be observed that the results obtained for both matrices do not completely overlap. The new alternative matrix is follicular fluid, which is innovative in case of PCOS research, especially in terms of oocytes maturation and their quality [21,22,23].

## 2. Metabolic Alterations in PCOS

PCOS includes a number of abnormalities, which influence several metabolic pathways. It is especially characterized by disturbed metabolism of the steroid hormones, amino acids, carbohydrates, lipids, purines, and the citric acid cycle. Searching for these pathological changes is possible through the metabolomic analyses of biological samples such as serum or plasma, urine, and follicular fluid. Most studies focus on serum and plasma analysis; however, other biological samples also provide substantial information on the existing biochemical derangements. In this review paper, we concentrated on metabolomic studies that were performed in the period of 2014 to 2020 and analysed different biological samples. The PubMed database was searched using the terms “polycystic ovary syndrome” or “PCOS” and “metabolomics”. The most important and actual studies using plasma, serum, urine, or FF samples were then analysed.

### 2.1. Metabolomic Profile Plasma and Serum Samples

Murri et al. (2014) published a valuable review where they compared few studies based on the analyses of plasma obtained from PCOS women and healthy controls [11,24,25,26,27]. In this paper, we quoted this publication and also presented the results of new studies in this field published since 2014 [28,29,30,31,32,33,34,35,36,37]. A set of metabolites found as the most characteristic for PCOS is presented in Table 1. Additionally, information about the applied technique as well as the trend of regulation is included. As can be observed, three metabolic pathways seem to characterise PCOS. Among them, metabolites connected with lipid, amino acid, as well as energy metabolism such as citric acid cycle seem to be the most common. In the case of PCOS, down-regulation of glycerophospholipid metabolism and up-regulation of glucose metabolism was observed. The results published by Zhao et al. (2012) show that all of the determined fatty acids are up-regulated in PCOS compared to the control subjects [25]. A contrary phenomenon occurs in the case of phosphatidylcholine (PC), phosphatidylethanolamine (PE) and its derivatives lysophosphatidylcholine (LPC), lysophosphatidylethanolamine (LPE). Metabolites including PC, PE, LPE, and LPC are decreased. In turn, Fan et al. (2019) observed a decreased level of compounds involved in the metabolism of lecithin [36]. In several studies, androgen metabolism was also taken into account. Three major metabolites connected with elevated androgen metabolism were found, namely dehydroepiandrosterone sulphate (DHEAS), dihydrotestosterone sulphate (DHTS), and androsterone sulphate (ANDS) (Table 1). Amino acids (AAs) are the next group of endogenous compounds determined in samples collected from women with PCOS. According to the presented database, there is no homogenous pattern in AAs’ regulation. For instance, the levels of arginine, choline, citrulline, glutamate, glycine, and histidine were found to be decreased. In turn, Zhao et al. (2012) reported increased levels of endogenous AAs and glucogenic AAs [25].

### 2.2. Metabolomic Profile of the Urine Samples

There are relatively few metabolomic studies on PCOS where urine is used as the biological matrix. Urine samples are a convenient study material due to non-invasive sampling as well as easy sample preparation because of the lower content of protein compared to serum and plasma [38]. This matrix is also rich in metabolites of the metabolic pathways, which may be deranged in PCOS. Metabolites, which were down- and up-regulated in women with PCOS compared to healthy controls, are presented in Table 2 [39,40,41]. Zou et al. (2018) reported that some carbohydrates and fatty acids metabolites are up-regulated in PCOS in comparison with the control subjects [40]. A contrary phenomenon occurs in the case of glycerolipids, where levels of 5 out of 7 compounds are decreased, while up-regulation of triglyceride (TG) and DG (16:1(9Z)/14:0/0:0) was reported. Dhayat et al. (2018) focused on the determination of androgens in PCOS, which were all elevated [41]. A similar trend is reported for AAs, glucocorticoids, and peptides.

### 2.3. Metabolomic Profile of Follicular Fluid Samples

Follicular fluid (FF) is an alternative and a useful biological matrix to study the potential mechanism of PCOS pathophysiology. FF is the product of plasma modified by the secretory activity of the granulosa and theca cells [42]. This matrix is collected from women with PCOS undergoing in vitro fertilization. FF contains metabolites essential for oocyte growth and maturation. The analysis of the metabolic derangements in FF samples from women with PCOS allows to understand pathological changes and also disclose the metabolites that could potentially disturb normal oocyte growth [43].

We reviewed a few metabolomic studies that analysed FF from women with PCOS and compared them with healthy controls [44,45,46,47,48]. They were performed with the use of different metabolomic techniques. As can be observed, the concentration of metabolites involved in the TCA cycle, as well as *α*-keto acids, are relatively higher in samples obtained from women with PCOS in comparison with the control subjects. The contrary trend was shown for acylcarnitines. For all of the determined metabolites belonging to acylcarnitines, the decreased level was determined for samples obtained from PCOS patients. However, there are a few metabolic pathways where the trend is not similar among the pathway, but is specific for individual subgroups of compounds. For example, as was reported by Liu et al. (2018), the decreased level of PC was observed in PCOS patients, while the contrary trend (up-regulation) was shown for Lyso PC [46]. In the case of fatty acyls, the general direction demonstrates down-regulation. However, Liu et al. (2018) reported an increased level of 1-Hydroxy-2,12,15-heneicosatrien-4-one in PCOS patients in comparison with the control subjects. The diversity was also observed in the case of AAs metabolism. For example, increased level of phenylalanine, valine, and isoleucine was observed, while a decreased amount of alanine, glutamine, and tyrosine was reported. The results are presented in Table 3.

## 3. Discussion

The summary of all the metabolites detected in three different biological matrices gives us a complementary overview of the metabolomic profile of women with PCOS and allows us to look for a correlation between altered levels of metabolites from different biochemical pathways.

Lipids are the largest group of molecules whose metabolism is deranged in women with PCOS. They are involved in various metabolic pathways, such as steroid hormone biosynthesis, sphingolipid, and fatty acids metabolisms like oxidation or amide metabolism. Phospholipids, which take part in multiple biological pathways, are down-regulated in PCOS. There are few studies confirming the decreased levels of sphinganine, LPE, especially LPE (22:5) and LPC, mainly LPC (18:2) in women with PCOS [27,28,34]. LPCs are involved in glucose metabolism, and low levels of LPC (18:2) correlate with IR and increased risk of T2DM. Thus, this would be in accordance with the observation that women with PCOS are more prone to these metabolic alterations [34]. Hauola et al. (2015) noticed that the differences in the lipid profiles between PCOS and healthy women correlate with the phase of the menstrual cycle. The most significant changes were detected between samples collected from healthy women during the luteal phase of the menstrual cycle and women with PCOS [35]. Among sphingolipids, phytosphingosine (PHS), which stimulates the transcriptional activity of the peroxisome proliferator-activated receptor γ (PPARγ), is downregulated in women with PCOS. According to the literature, PPARs are ligand-dependent transcription factors that regulate the expression of numerous genes associated with the metabolism of carbohydrates, lipids, and proteins [30]. Dysfunction of these receptors may also contribute to the increased risk of MetS and T2DM. The latest lipidomic studies indicate that elevated levels of DG and cholesterol ester, and lower levels of LysoPC correlate with IR, irrespective of a person being overweight or obese [49].

Szczuko et al. (2017) analysed plasma fatty acids in women with PCOS. Their results showed that the levels of all free fatty acids were lower in women with PCOS; however, the concentration of nervonic acid was several times (almost 330 times) higher than in the control subjects. This level of nervonic acid was observed in two analysed groups of women, namely women with PCOS with a biochemical indication of hyperandrogenism and women with normal androgen levels. Poly-unsaturated fatty acids (PUFAs), the precursors of eicosanoids, were significantly increased in women with PCOS compared with the control subjects, which may be due to the presence of low-grade systemic inflammation. In recent years, some studies reported that this process could be stimulated by the pro-inflammatory interleukin-1 (IL-1), which is overexpressed in women with PCOS [50].

One of the detected metabolites, which may be considered as a potential biomarker of PCOS, is DHEA. Excess levels of DHEA are mostly detected in the metabolome of women with PCOS [29,30,33,34]. Serum concentrations of DHEA-S are also evaluated in clinical practice for the evaluation of biochemical hyperandrogenism in women. Besides, 19-oxotestosterone levels are also elevated in women with POCS. This could be due to the higher activity of aromatase, which catalyses the formation of C18 estrogens from C19 androgens [30]. Zhao et al. (2014) showed that, apart from elevated DHEA levels in women with PCOS, there is a significant increase of DHTS and ANDS, which points to the exaggerated androgen synthesis [29]. Dhayat et al. (2018) reported that in women with PCOS, there is an alternative pathway of 11-oxygenated androgen production. They also reported that four steroids such as androstanediol, estriol, 20-β-dihydrocortisone, and cortisol are found as potential markers of PCOS [41].

Lipidomic analysis was also the main goal of the study conducted by Vonica et al. (2019). The authors reported that alterations in acylglycerols, PGs and LTs, phosphocholines, and carnitine metabolites occur in women with PCOS. As a result, cholestane-5α (18:1/0:0), triacylglycerol (18:2/18:2/0-18:0), cholestane-3β, 5α, 6β-triol (18:0/0:0) were found as the crucial metabolites to identify women with PCOS from the controls. Serum levels of these metabolites were decreased. Moreover, elevated acylcarnitine: 2-hydroxylauroylcarnitine with decreased phosphocholines metabolites (18:1/18:4, 18:3/18:2) were also observed. The authors assumed that this alteration might be linked to the lipid peroxidation. Levels of some of the TG (18:2/18:2/0-18:0) and DG species (18:1/20:0/0:0, 18:0/0:0/20:1, 22:4n6/0:0/18:4n3, 18:3/0:0/22:5, 20:0/0:0/20:0, 18:1/24:0/0:0, 18:0/0:0/24:1) were also elevated in women with PCOS in comparison with the control subjects [37]. This can be explained by a higher BMI and increased intra-abdominal fat deposition, which is also a hallmark of PCOS. Dyslipidemia, which occurs in PCOS is characterized by the increased concentrations of total cholesterol (TCh), low density lipoproteins (LDL), very-low density lipoproteins (VLDL), and TGs coupled with decreased high-density lipoproteins (HDL) and HDL-cholesterol. During intestinal absorption, TGs are degraded to fatty acids. After that, they are again resynthesized and transported by the chylomicrons through the bloodstream to the adipose and muscle tissue, where their degradation into free fatty acids and monoacylglycerol takes place. Elevated levels of TGs in PCOS women may be caused by a high fat intake or reduced fat energy consumption at night. It was confirmed that in women with PCOS, the reduced ability to switch to lipid oxidation during fasting also occurs at night [37].

7β-Hydroxycholesterol is another lipid metabolite that could also be a potential biomarker of PCOS. It highly correlates with a PCOS diagnosis. This oxysterol is found as a metabolic intermediate or the end-product of cholesterol metabolism. Due to its bioactivity, it could induce oxidative stress and disturb the metabolism of fatty acids. Chen et al. (2020) showed that elevated levels of 7β-hydroxycholesterol measured in the FF by the induction of the oxidative stress may disturb the growth of oocytes in PCOS [48].

There also are some specific alterations of the bile acid metabolism. These abnormalities and dysfunction of fat absorption in women with PCOS is due to the decreased level of glycocholic acid, which was reported by Zhao et al. (2014) [29] and confirmed by Jia et al. (2019) [34].

The metabolism of prostaglandins and carnitine also seems to be deranged in women with PCOS. Vonica et al. (2019) reported an increased level of the prostaglandin (PG) E2 pathway and oxo-leukotrienes (LT) known to play a pivotal role in inflammation [37]. Dong et al. (2015) showed an increase of prostaglandin F2a (FPG-2a) and a decrease of l-carnitine in women with PCOS [30]. Elevated levels of FPG-2a may also be related to the presence of a low-grade systemic inflammation in PCOS. In turn, l-carnitine plays an essential role in fatty acid metabolism as well as their transport across the mitochondrial membrane to the mitochondrial matrix where β-oxidation of fatty acids (FAs) occurs. Decreased levels of l-carnitine may point to the impairment of these processes in PCOS. It could result in an accumulation of the FAs in the cytosol. It is noticed that l-carnitine supplementation may improve oocyte quality and therefore, may have a positive effect on fertility [30]. Carnitine is also involved in stabilizing acetylCoA and coenzyme A levels and plays an essential role during fetal maturation [34]. Elevated levels of acetate found by Whigham et al. (2014) and RoyChodhury et al. (2016) also indicate reduced FAs oxidation in women with PCOS [28].

**Figure 2:**
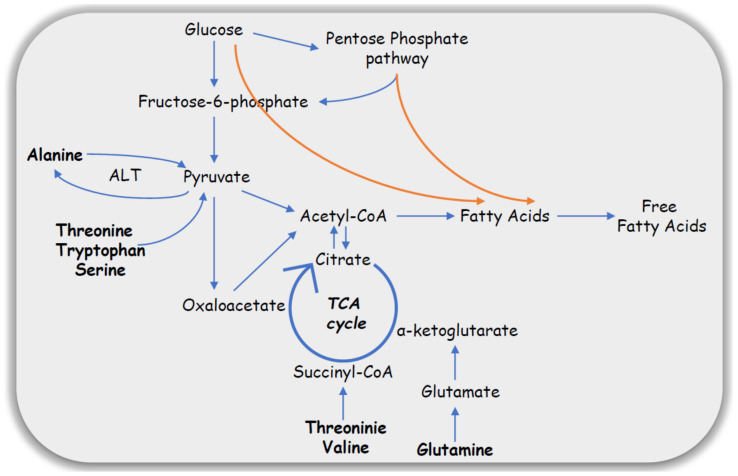
Alteration of glucose metabolism in PCOS.

Impaired carbohydrate metabolism is a hallmark of PCOS. Elevated serum lactate and reduced level of glucose determined in plasma samples suggest alterations in glucose metabolism accompanied with elevated glycolytic activity associated with the TCA cycle impairment. Whigham et al. (2014) suggested that in women with PCOS, some AAs are utilized as a source of alternative energy metabolism [24]. Alanine is an important amino acid in the process of gluconeogenesis. Serum alanine-amino transaminase (ALT) activity is elevated in women with PCOS, which usually is the result of NAFLD and lead to increased alanine transformation [27]. According to the literature, ALT is involved in urea cycle and AAs metabolism, where alanine may be converted to pyruvate by donating an amine group and enter the TCA cycle as well as be formed from pyruvate by accepting an amine group, respectively. Serum levels of glucogenic AAs acids such as valine, leucine, and threonine, which may also enter the TCA cycle or be involved in the process of gluconeogenesis were also significantly elevated in women with PCOS. The higher number of growing antral follicles in PCOS utilizes more energy. For this purpose, carbohydrate metabolism may not be sufficient and may thus lead to the utilization of other energy substrates like glutamine, glutamate, or 3-hydroxybutyric acid. Whigham et al. (2014) pointed out that the TCA cycle and glucose metabolism are the major pathways deranged in PCOS [28]. As reported, glucose metabolism can be carried out via alternative pathways such as glycolysis or pentose phosphate pathways and lead to an increased FAs synthesis, which may explain the observed increased FAs accumulation in the adipocytes (Figure 2).

In women with PCOS elevated levels of phenylalanine and glycated phenylalanine were also detected. The accumulation of the glycated AAs was also reported in T2DM [25]. The lower level of other AAs such as proline and histidine may be due to the increased utilization of these AAs as antioxidants during the oxidative stress present in PCOS.

Vitamin B6 metabolism may also be deranged in women with PCOS. One of its metabolic pathways is the synthesis and degradation of the AAs. The results of Chen et al. (2020) showed a significant increase of pyridoxal 5′-phosphate (PLP) and d-glutamic acid in the FF of women with PCOS. The authors claimed that both compounds are linked with the vitamin B6 metabolism. On the other hand, it is known that PLP is a coenzyme in the metabolism of homocysteine. Moreover, increased levels of homocysteine were observed in women with PCOS. Taking this into the account, disruption of the homocysteine metabolism in PCOS may impair the oocyte microenvironment. The second metabolite detected by the authors was glutamic acid. Its levels are strictly associated with efficiency of the glutamate decarboxylase, while the activity of this enzyme is regulated by the presence of vitamin B6. Glutamic acid is essential for the growth of oocytes, because it can be utilized as an alternative source of energy. These authors also suggested that the increased level of this metabolite is due to its accumulation in the FF [48].

Tang et al. (2019) pointed out that amino acid metabolic abnormalities are also characteristic of PCOS [51]. Among the branched-chain AAs, three were pointed out: valine, leucine, and isoleucine. All of them were up-regulated in women with PCOS. It is assumed that increased levels of valine, leucine, and isoleucine may affect the progression of the IR and obesity. From a biological point of view, branched-chain AAs can serve as substrates for the synthesis of glucose. It occurs in the case of IR, where abnormal glucose metabolism is carried out and alanine is obtained by the transamination of pyruvic acid. Second subgroup of AAs, which are associated with IR are lysine, phenylalanine, and 2-aminoadipic acid. Studies conducted by Tang et al. (2019) pointed to significantly higher concentrations of these metabolites in women with PCOS, which can also be associated with the IR [51]. Chen et al. (2020) reported that phenylalanine may be important for the growth and development of oocytes and could therefore be associated with ovulatory dysfunction [48].

IR plays a key role in the pathophysiology of PCOS and is associated with ovulatory dysfunction and hyperandrogenism. Some mechanisms of IR in women with PCOS are connected to an excessive activity of 17 α-hydroxylase, which regulates the conversion of 17-hydroxyprogesterone into androstenedione, excessive stimulation of IGF-I receptors, and diminished synthesis of insulin-like growth factor binding protein 1 (IGF-BP1) [52]. 

The Rotterdam diagnostic criteria yield four separate PCOS phenotypes (A, B, C, and D). Phenotype A includes all the three features (HA, AnO, and PCOM), whereas phenotype B, C, and D include only two (HA and AnO or HA and PCOM or AnO and PCOM, respectively). Among the mentioned PCOS studies, only one analyzed the metabolites among four different PCOS phenotypes (A, B, C, and D). Zhao et al. (2012) reported elevated levels of leucine and decreased levels of serine and threonine in women with the C (HA + PCOM) phenotype in comparison to the other phenotypes [25].

### Limitations of Metabolomic Studies

The range of described metabolites identified during metabolomic studies is enormous and gives an overview of the metabolic profile of women with PCOS. Several different compounds and many biochemical pathways seem to be involved in the pathogenesis of PCOS, which indicates the complexity of this common endocrinopathy. These alterations may be caused by an increased or reduced efficiency of different biochemical reactions, up- or down-regulation of genes, increased or decreased activity of enzymes, as well the formation of alternative metabolic pathways. Metabolomics enables us to study these biochemical pathways, which might be involved in the pathogenesis of PCOS. However, it is important to remember that there are some limitations of metabolomic studies. An enormous challenge in research, which is performed with the use of human matrices is inter-individual variability, especially in women, in whom the range of the detected metabolites could be correlated to different hormone levels during the menstrual cycle. Some metabolomic studies were performed relatively in a small group of women, with only tentatively identified metabolites. There could also be some difficulties with the efficiency of the analysers, which sometimes yield false positive results. Furthermore, differences in sample preparation also have a significant impact on the final results.

Therefore, in metabolomics research, every step of the study is significant—from appropriate patient requirements through analytical accuracy to the identification and statistical analysis of the obtained results. That is why there is the need to confirm if the detected compounds could become reliable biomarkers, which would selectively distinguish PCOS from other endocrinopathies.

## 4. Conclusions

**Figure 3:**
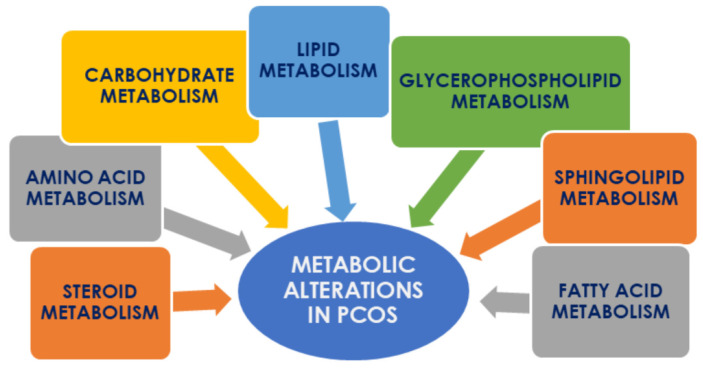
The main biochemical pathways disturbed in PCOS.

Metabolomics has proven to be a potential tool in studying the pathophysiology of PCOS. The application of metabolomics allows us to discover metabolic pathways that have been shown to be deranged. These abnormalities are associated mainly with the metabolism of lipids, fatty acids, sphingolipids and glycerophospholipids, steroids as well as carbohydrates and amino acids (Figure 3). Additionally, some alternative biochemical processes have been shown to be up-regulated in women with PCOS; however, their clinical significance should be confirmed and evaluated. Determination of disturbed pathways allows identification of the specific compounds characteristic of PCOS, which might be considered as biomarkers and became potential targets for future metabolomic research. Finding appropriate biochemical markers could be a milestone in early diagnosis of this endocrinopathy and a starting point for targeted future pharmacological interventions.

## Figures and Tables

**Table 1:** The most significant changes in metabolites measured in plasma and serum samples in women with PCOS in comparison with control subjects.

Metabolites	PCOS vs. Control	Metabolic Pathways	Studies	Techniques
Cholesterol	↓	Lipid metabolism	Zhao et al., 2012	GC-MS GC-MS
	↓		Escobar-Morreale et al., 2012	
	↓		Buszewska-Forajta et al., 2019	
Alpha-Tocopherol	↓	Lipid metabolism	Escobar-Morreale et al., 2012	GC-MS
HDL	↓	Lipid metabolism	Zhao et al., 2012	NMR
Phosphatidylcholine	↓	Lipid metabolism	Zhao et al., 2012	NMR
	↓		Sun et al., 2012	NMR
Linoleic acid	↑	Lipid metabolism	Zhao et al., 2012	GC-MS
	↑		Dong et al., 2015	LC-MS
Lipoprotein	↑	Lipid metabolism	Zhao et al., 2012	NMR
Palmitic acid	↑	Lipid metabolism	Zhao et al., 2012	GC-MS
C18:0 stearic acid	↑	Lipid metabolism	Zhao et al., 2012	GC-MS
	↓		Szczuko et al., 2017	GC-MS
Unsaturated fatty acid	↑	Lipid metabolism	Zhao et al., 2012	NMR
VLDL/LDL	↑	Lipid metabolism	Zhao et al., 2012	NMR
Lipid-CH2CH2CO	↓	Lipid metabolism	Atiomo et al., 2012	NMR
	↑		Zhao et al., 2012	NMR
FFA 16:1	↑	Lipid metabolism	Zhao et al., 2014	LC-MS
FFA 16:2	↑	Lipid metabolism	Zhao et al., 2014	LC-MS
FFA 18:1	↑	Lipid metabolism	Zhao et al., 2014	LC-MS
FFA 18:3	↑	Lipid metabolism	Zhao et al., 2014	LC-MS
FFA 20:1	↑	Lipid metabolism	Zhao et al., 2014	LC-MS
FFA 20:2	↑	Lipid metabolism	Zhao et al., 2014	LC-MS
FFA 20:3	↑	Lipid metabolism	Zhao et al., 2014	LC-MS
FFA 20:4	↑	Lipid metabolism	Zhao et al., 2014	LC-MS
FFA 20:5	↑	Lipid metabolism	Zhao et al., 2014	LC-MS
FFA 20:6	↑	Lipid metabolism	Zhao et al., 2014	LC-MS
FFA 22:5	↑	Lipid metabolism	Zhao et al., 2014	LC-MS
FFA 22:6	↑	Lipid metabolism	Zhao et al., 2014	LC-MS
FFA 24:2	↑	Lipid metabolism	Zhao et al., 2014	LC-MS
MG 18:1	↑	Lipid metabolism	Zhao et al., 2014	LC-MS
MG 20:3	↑	Lipid metabolism	Zhao et al., 2014	LC-MS
LPC (16:1)	↓	Lipid metabolism	Zhao et al., 2014	LC-MS
LPC (16:0)	↓	Lipid metabolism	Haoula et al., 2015	LC-MS
LPC (18:0)	↓	Lipid metabolism	Haoula et al., 2015	LC-MS
LPC (18:1)	↓	Lipid metabolism	Zhao et al., 2014	LC-MS
	↓		Haoula et al., 2015	LC-MS
LPC (18:2)	↓	Lipid metabolism	Zhao et al., 2014	LC-MS
	↓		Dong et al., 2015	LC-MS
	↓		Jia et al., 2019	LC-MS
	↑		Buszewska-Forajta et al., 2019	LC-MS
	↓		Haoula et al., 2015	LC-MS
LPC 18:3	↓	Lipid metabolism	Zhao et al., 2014	LC-MS
	↓		Dong et al., 2015	LC-MS
LPC 20:5	↓	Lipid metabolism	Zhao et al., 2014	LC-MS
LPC 22:5	↓	Lipid metabolism	Zhao et al., 2014	LC-MS
LPE 16:0	↓	Lipid metabolism	Zhao et al., 2014	LC-MS
LPE 18:1	↓	Lipid metabolism	Zhao et al., 2014	LC-MS
LPE 18:2	↓	Lipid metabolism	Zhao et al., 2014	LC-MS
LPE 20:4	↓	Lipid metabolism	Zhao et al., 2014	LC-MS
LPE 22:5	↓	Lipid metabolism	Zhao et al., 2014	LC-MS
	↓		Dong et al., 2015	LC-MS
	↓		Jia et al., 2019	LC-MS
PC (18:1/18:4)	↓	Lipid metabolism	Vonica et al., 2019	LC-MS
PC (18:3/18:2)	↓	Lipid metabolism	Vonica et al., 2019	LC-MS
PC (32:4)	↓	Lipid metabolism	Haoula et al., 2015	LC-MS
PC (30:0)	↓	Lipid metabolism	Haoula et al., 2015	LC-MS
PE (42:1)	↓	Lipid metabolism	Haoula et al., 2015	LC-MS
PE (34:0)	↓	Lipid metabolism	Haoula et al., 2015	LC-MS
SM (d18:0/20:2)	↑	Lipid metabolism	Haoula et al., 2015	LC-MS
SM (d18:0/18:0)	↑	Lipid metabolism	Haoula et al., 2015	LC-MS
Triglycerides	↑	Lipid metabolism	Haoula et al., 2015	LC-MS
DG (36:2)	↑	Lipid metabolism	Haoula et al., 2015	LC-MS
DG (36:3)	↑	Lipid metabolism	Haoula et al., 2015	LC-MS
Plasmalogen (30:0)	↓	Lipid metabolism	Haoula et al., 2015	LC-MS
Plasmalogen (40:7)	↑	Lipid metabolism	Haoula et al., 2015	LC-MS
Azelaic acid	↑	Lipid metabolism	Dong et al., 2015	LC-MS
N-undecanoylglycine	↑	Lipid metabolism	Dong et al., 2015	LC-MS
Chenodeoxycholic acid	↑	Lipid metabolism	Fan et al., 2019	LC-MS
Cholic acid	↓	Lipid metabolism	Fan et al., 2019	LC-MS
Clupanodonylcarnitine	↑	Lipid metabolism	Fan et al., 2019	LC-MS
2-Hydroxylauroylcarnitine	↑	Lipid metabolism	Vonica et al., 2019	LC-MS
Trans-2-dodecenoylcarnitine	↑	Lipid metabolism	Vonica et al., 2019	LC-MS
Cholestane-3β	↑	Sterol lipid metabolism	Vonica et al., 2019	LC-MS
Cholestane-5α (18:0/0:0)	↑	Sterol lipid metabolism	Vonica et al., 2019	LC-MS
Cholestane-6β-triol	↑	Sterol lipid metabolism	Vonica et al., 2019	LC-MS
Cholestane (18:1/0:0)	↑	Sterol lipid metabolism	Vonica et al., 2019	LC-MS
Androsterone sulphate	↑	Lipid transport and metabolism	Fan et al., 2019	LC-MS
11′-Carboxy-α-chromanol	↑	Lipid transport and metabolism	Fan et al., 2019	LC-MS
(9-cis,9′-cis)-7,7′,8,8′-Tetrahydro-y,y-Carotene	↑	Lipid transport and metabolism	Fan et al., 2019	LC-MS
Sphinganine	↓	Sphingolipid metabolism	Dong et al., 2015	LC-MS
	↓		Jia et al., 2019	LC-MS
	↑		Buszewska-Forajta et al., 2019	LC-MS
Phytosphingosine	↓	Sphingolipid metabolism	Dong et al., 2015	LC-MS
Palmitoylsphingomyelin	↑	Sphingomyelin metabolism	Fan et al., 2019	LC-MS
SM (d18:1/16:0)	↑	Sphingomyelin metabolism	Fan et al., 2019	LC-MS
LysoPC (O-18:0)	↓	Lecithin metabolism	Fan et al., 2019	LC-MS
LysoPC (16:0)	↓	Lecithin metabolism	Fan et al., 2019	LC-MS
LysoPC [20:2(11Z,14Z)]	↓	Lecithin metabolism	Fan et al., 2019	LC-MS
Glyceric acid	↑	Glycerolipid metabolism	Dong et al., 2015	LC-MS
LPC (20:2)	↓	Glycerophospholipid metabolism	Dong et al., 2015	LC-MS
2-Arachidonoyl	↑	Glycerophospholipid metabolism	Fan et al., 2019	LC-MS
glycerophosphocholine				
PG [18:1(9Z)/16:0]	↓	Glycerophospholipid metabolism	Fan et al., 2019	LC-MS
PE [O-18:1(1Z)/20:4 (5Z,8Z,11Z,14Z)]	↓	Glycerophospholipid metabolism	Fan et al., 2019	LC-MS
LysoPE [0:0/22:1(13Z)]	↓	Glycerophospholipid metabolism	Fan et al., 2019	LC-MS
PE [O-16:1(1Z)/22:6 (4Z,7Z,10Z,13Z,16Z,19Z)]	↓	Glycerophospholipid metabolism	Fan et al., 2019	LC-MS
PE [22:4(7Z,10Z,13Z,16Z)/16:0]	↓	Glycerophospholipid metabolism	Fan et al., 2019	LC-MS
PC [16:1(9Z)/22:2(13Z,16Z)]	↓	Glycerophospholipid metabolism	Fan et al., 2019	LC-MS
PG (18:0/16:0)	↑	Glycerophospholipid metabolism	Fan et al., 2019	LC-MS
PG (18:1(9Z)/18:0)	↓	Glycerophospholipid metabolism	Fan et al., 2019	LC-MS
DG (18:1n9/0:0/20:4n3)	↑	Diacyloglycerol metabolism	Fan et al., 2019	LC-MS
TG (18:2/18:2/0-18:0)	↑	Diacyloglycerol metabolism	Vonica et al., 2019	LC-MS
DG (22:2/0:0/22:4)	↓	Diacyloglycerol metabolism	Vonica et al., 2019	LC-MS
Arginine	↓	Amino acids metabolism	Atiomo et al., 2012	NMR
	↓		Sun et al., 2012	NMR
Choline	↓	Amino acids metabolism	Sun et al., 2012	NMR
Citruline	↓	Amino acids metabolism	Atiomo et al., 2012	NMR
Glutamate	↓	Amino acids metabolism	Atiomo et al., 2012	NMR
Glycerophosphocholine/phosphocholine	↓	Amino acids metabolism	Sun et al., 2012	NMR
Glycine	↓	Amino acids metabolism	Zhao et al., 2012	GC-MS
Histidine	↓	Amino acids metabolism	Atiomo et al., 2012	NMR
	↓		RoyChoudhury et al., 2016	
AAA	↑	Amino acids metabolism	Zhao et al., 2012	GC-MS
BCAA	↑	Amino acids metabolism	Zhao et al., 2012	GC-MS
BCAA/AAA	↓	Amino acids metabolism	Zhao et al., 2012	GC-MS
Aspartate	↑	Amino acids metabolism	Zhao et al., 2012	GC-MS
Endogenous AAs	↑	Amino acids metabolism	Zhao et al., 2012	GC-MS
Gluconeogenic AAs	↑	Amino acids metabolism	Zhao et al., 2012	GC-MS
Serine	↑	Amino acids metabolism	Zhao et al., 2012	GC-MS
2-Aminobutyrate	↑	Amino acid metabolism	Whigham et al., 2014	NMR
2-Hydroxybutyrate	↑	Amino acid metabolism	Whigham et al., 2014	NMR
2-Hyroxyisovalerate	↑	Amino acid metabolism	Whigham et al., 2014	NMR
2-Oxocaproate	↑	Amino acid metabolism	Whigham et al., 2014	NMR
2-Oxoisocaproate	↑	Amino acid metabolism	Whigham et al., 2014	NMR
3-Hydroxybutyrate	↑	Amino acid metabolism	Whigham et al., 2014	NMR
3-Methyl-2-oxovalerate	↑	Amino acid metabolism	Whigham et al., 2014	NMR
Betadine	↑	Amino acid metabolism	Whigham et al., 2014	NMR
Creatinine	↑	Amino acid metabolism	Whigham et al., 2014	NMR
	↓		Sun et al., 2012	NMR
Dimethylamine	↑	Amino acid metabolism	Whigham et al., 2014	NMR
Lysine	↑	Amino acid metabolism	Whigham et al., 2014	NMR
	↑		Zhao et al., 2012	GC-MS
	↓		Atiomo et al., 2012	NMR
Methionine	↑	Amino acid metabolism	Whigham et al., 2014	NMR
	↓		Sun et al., 2012	NMR
Ornithine	↑	Amino acid metabolism	Whigham et al., 2014	NMR
	↑		Zhao et al., 2012	GC-MS
	↓		Atiomo et al., 2012	NMR
Sarcosine	↑	Amino acid metabolism	Whigham et al., 2014	NMR
Taurine	↑	Amino acid metabolism	Whigham et al., 2014	NMR
Tryptophan	↑	Amino acid metabolism	Whigham et al., 2014	NMR
	↑		Zhao et al., 2012	GC-MS
	↑		Buszewska-Forajta et al., 2019	GC/LC-MS
Tyrosine	↑	Amino acid metabolism	Whigham et al., 2014	NMR
	↑		Zhao et al., 2012	GC-MS
	↑		Buszewska-Forajta et al., 2019	GC-MS
Glutamate	↓	Amino acids metabolism	RoyChoudhury et al., 2016	NMR
	↑		Whigham et al., 2014	NMR
Glutamine	↓	Amino acids metabolism	RoyChoudhury et al., 2016	NMR
	↑		Whigham et al., 2014	NMR
	↓		Sun et al., 2012	NMR
Proline	↓	Amino acids metabolism	Atiomo et al., 2012	NMR
	↓		Zhao et al., 2012	GC-MS
	↓		RoyChoudhury et al., 2016	NMR
	↑		Whigham et al., 2014	NMR
Alanine	↑	Amino acids metabolism	RoyChoudhury et al., 2016	NMR
	↑		Whigham et al., 2014	NMR
	↑		Zhao et al., 2012	NMR
	↓		Escobar-Morreale et al., 2012	GC-MS
Leucine	↑	Amino acids metabolism	RoyChoudhury et al., 2016	NMR
	↑		Whigham et al., 2014	NMR
	↓		Sun et al., 2012	NMR
	↑		Zhao et al., 2012	GC-MS
Isoleucine	↑	Amino acids metabolism	Whigham et al., 2014	NMR
	↓		Zhao et al., 2012	GC-MS
Valine	↑	Amino acids metabolism	RoyChoudhury et al., 2016	NMR
	↑		Whigham et al., 2014	NMR
	↑		Zhao et al., 2012	GC-MS
Threonine	↑	Amino acids metabolism	RoyChoudhury et al., 2016	NMR
	↑		Whigham et al., 2014	NMR
	↑		Zhao et al., 2012	GC-MS
	↑		Buszewska-Forajta et al., 2019	GC-MS
Cysteine-S-sulphate	↑	Amino acid metabolism	Fan et al., 2019	LC-MS
Glu-Glu	↑	Amino acid metabolism	Dong et al., 2015	LC-MS
Asparagine	↑	Amino acid metabolism	Whigham et al., 2014	NMR
Ketoleucine	↓	Valine, leucine, and isoleucine degradation	Dong et al., 2015	LC-MS
Glutamic acidc	↑	Glutamate metabolism, amino sugar metabolism	Dong et al., 2015	LC-MS
Phenylpyruvic acid	↑	Phenylalanine and tyrosine metabolism	Dong et al., 2015	LC-MS
Gly.Phe	↑	Phenylalanine and tyrosine metabolism	Zhao et al., 2014	LC-MS
Phenylalanine	↑	Phenylalanine and tyrosine metabolism	Zhao et al., 2014	LC-MS
	↑		Whigham et al., 2014	NMR
	↑		Zhao et al., 2012	GC-MS
	↑		Buszewska-Forajta et al., 2019	GC-MS
Phe−Phe	↑	Phenylalanine and tyrosine metabolism	Zhao et al., 2014	LC-MS
Kynurenine	↓	Tryptophan metabolism	Zhao et al., 2014	LC-MS
5-Hydroxyindoleacetic acid	↓	Tryptophan metabolism	Dong et al., 2015	LC-MS
Homoserine	↓	Methionine metabolism	Zhao et al., 2014	LC-MS
	↑		Whigham et al., 2014	NMR
S-Adenosylmethionine	↓	Thiol amino acid metabolic cycle	Fan et al., 2019	LC-MS
Pyroglutamic acid	↑	Glutathione metabolism	Dong et al., 2015	LC-MS
Lysyl-albumin	↓	Protein metabolism	Zhao et al., 2012	NMR
Trimethylamine N-oxide	↓	Protein metabolism	Sun et al., 2012	NMR
2-Ketoisocaproic acid	↓	Protein metabolism	Escobar-Morreale et al., 2012	GC-MS
Dimethylamine	↑	Protein metabolism	Sun et al., 2012	NMR
*N*-acetylglycoprotein	↓	Protein metabolism	Zhao et al., 2012	NMR
	↑		Sun et al., 2012	NMR
Hypoxanthine	↑	Purine metabolism	Zhao et al., 2014	LC-MS
Inosine	↑	Purine metabolism	Zhao et al., 2014	LC-MS
Allantoic acid	↑	Purine metabolism	Dong et al., 2015	LC-MS
Uric acid	↑	Purine metabolism	Zhao et al., 2012	GC-MS
	↑		Buszewska-Forajta et al., 2019	GC/LC-MS
Cyclic GMP	↑	Purine metabolism	Fan et al., 2019	LC-MS
Uridine	↓	Pyrimidine metabolism	Zhao et al., 2014	LC-MS
	↓		Dong et al., 2015	LC-MS
5,6-Dihydrouridine	↑	Pyrimidine metabolic cycle	Fan et al., 2019	LC-MS
DHEAS	↑	Androgen metabolism	Zhao et al., 2014	LC-MS
	↑		Dong et al., 2015	LC-MS
	↑		Buszewska-Forajta et al., 2019	LC-MS
	↑		Jia et al., 2019	LC-MS
	↑		Fan et al., 2019	LC-MS
ANDS	↑	Androgen metabolism	Zhao et al., 2014	LC-MS
DHTS	↑	Androgen metabolism	Zhao et al., 2014	LC-MS
Pregnenolone sulphate	↓	Steroid hormone biosynthesis	Dong et al., 2015	LC-MS
19-Oxotestosterone	↑	Steroid hormone biosynthesis	Dong et al., 2015	LC-MS
C10:0 lauric acid	↓	Fatty acid metabolism	Szczuko et al., 2017	GC-MS
C15:0 pentadecanoic acid	↓	Fatty acid metabolism	Szczuko et al., 2017	GC-MS
C15:1 cis-10-pentadecanoic acid	↓	Fatty acid metabolism	Szczuko et al., 2017	GC-MS
C17:0 heptadecanoic acid	↓	Fatty acid metabolism	Szczuko et al., 2017	GC-MS
C20:0 arachidic acid	↓	Fatty acid metabolism	Szczuko et al., 2017	GC-MS
C20:1 cis-11-eicosanoic acid	↑	Fatty acid metabolism	Szczuko et al., 2017	GC-MS
C22:5 EPA	↓	Fatty acid metabolism	Szczuko et al., 2017	GC-MS
C22:0 behenic acid	↓	Fatty acid metabolism	Szczuko et al., 2017	GC-MS
C23:0 tricosanoic acid	↓	Fatty acid metabolism	Szczuko et al., 2017	GC-MS
C22:4n6 docosatetraenic acid	↑	Fatty acid metabolism	Szczuko et al., 2017	GC-MS
C24:0 lignoceric acid	↓	Fatty acid metabolism	Szczuko et al., 2017	GC-MS
C24:1 nervonic acid	↑	Fatty acid metabolism	Szczuko et al., 2017	GC-MS
9-HODE/13-HODE	↑	Fatty acid metabolism	Dong et al., 2015	LC-MS
α-Linolenic acid	↑	Fatty acid metabolism	Dong et al., 2015	LC-MS
C18:2n6c linoleic acid	↓	Fatty acid metabolism	Szczuko et al., 2017	GC-MS
Vaccenic acid	↑	Fatty acid metabolism	Dong et al., 2015	LC-MS
Docosatrienoic acid	↑	Fatty acid metabolism	Dong et al., 2015	LC-MS
Eicosapentaenoic acid	↑	Fatty acid metabolism	Dong et al., 2015	LC-MS
Galbanic acid	↑	Fatty acid metabolism	Fan et al., 2019	LC-MS
C14:0 myristic acid	↑	Fatty acid biosynthesis	Dong et al., 2015	LC-MS
	↓		Szczuko et al., 2017	GC-MS
Palmitoleic acid	↑	Fatty acid biosynthesis	Dong et al., 2015	LC-MS
Palmitoleoylethanolamide	↑	Fatty acid amide metabolism	Dong et al., 2015	LC-MS
Oleamide	↑	Fatty acid amide metabolism	Zhao et al., 2014	LC-MS
	↑		Dong et al., 2015	LC-MS
Palmitic amide	↑	Fatty acid amide metabolism	Zhao et al., 2014	LC-MS
	↑		Dong et al., 2015	LC-MS
PEA	↑	Fatty acid amide metabolism	Zhao et al., 2014	LC-MS
AEA	↑	Fatty acid amide metabolism	Zhao et al., 2014	LC-MS
Carnitine C2:0	↑	Beta oxidation of fatty acids	Zhao et al., 2014	LC-MS
Carnitine C6:0	↑	Beta oxidation of fatty acids	Zhao et al., 2014	LC-MS
Carnitine C18	↑	Beta oxidation of fatty acids	Zhao et al., 2014	LC-MS
Carnitine	↓	Oxidation of fatty acids	Dong et al., 2015	LC-MS
	↓		Jia et al., 2019	LC-MS
Glycocholic acid	↓	Bile acid metabolism	Zhao et al., 2014	LC-MS
	↓		Jia et al., 2019	LC-MS
3,7-Dihydroxy-5-cholestenoic acid	↑	Bile acid metabolism	Fan et al., 2019	LC-MS
3-β-Hydroxy-4-β-methyl-5-α-cholest-7-ene-4-α-carboxylate	↑	Bile acid metabolism	Fan et al., 2019	LC-MS
Formate	↑	Pyruvate metabolism	Whigham et al., 2014	NMR
Fructose	↑	Pyruvate metabolism	Whigham et al., 2014	NMR
Mannose	↑	Pyruvate metabolism	Whigham et al., 2014	NMR
Citrate	↓	TCA cycle metabolism	Whigham et al., 2014	NMR
	↓		Atiomo et al., 2012	NMR
	↓		Sun et al., 2012	NMR
Acetate	↑	TCA cycle metabolism	RoyChoudhury et al., 2016	NMR
	↑		Whigham et al., 2014	NMR
4a-Methylzymosterol-4-carboxylic acid	↑	TCA cycle metabolism	Fan et al., 2019	LC-MS
Lactate	↑	Gluconeogenesis/Glycolysis	RoyChoudhury et al., 2016	NMR
	↑		Whigham et al., 2014	NMR
	↑		Zhao et al., 2012	GC-MS/
				NMR
Lactic acid	↑	Gluconeogenesis/Glycolysis	Buszewska-Forajta et al., 2019	GC-MS
Gluconolactone	↑	Pentose phosphate pathway	Dong et al., 2015	LC-MS
3-Hydroxybutyric acid	↓	Energy metabolism	RoyChoudhury et al., 2016	NMR
Glucose	↓	Energy metabolism	RoyChoudhury et al., 2016	NMR
	↓		Zhao et al., 2012	GC-MS
	↑		Whigham et al., 2014	/NMR
				NMR
Glyceraldehyde 3-phosphate	↑	ATP metabolism	Fan et al., 2019	LC-MS
Glycerol	↓	Glucose metabolism	Whigham et al., 2014	NMR
Acetoacetate	↑	Glucose metabolism	Whigham et al., 2014	NMR
Pyruvate	↑	Glucose metabolism	Whigham et al., 2014	NMR
Acetone	↑	Glucose metabolism	Whigham et al., 2014	NMR
	↓		Atiomo et al., 2012	NMR
Fructose 6-phosphate	↓	Amino sugar metabolism	Dong et al., 2015	LC-MS
Aspartic acid	↑	Aspartate metabolism	Zhao et al., 2014	LC-MS
Thyroxine sulphate	↓	ATP metabolism	Fan et al., 2019	LC-MS
Pantothenic acid	↑	Pantothenate and CoA biosynthesis	Dong et al., 2015	LC-MS
Prostaglandin F2a	↑	Arachidonic acid metabolism	Dong et al., 2015	LC-MS
	↑		Vonica et al., 2019	LC-MS
25-Methyl-1-hexacosanol	↓	Fatty alcohols	Fan et al., 2019	LC-MS
S-(PGJ2)—glutathione	↑	Immune modulation	Fan et al., 2019	LC-MS
Oryzanol A	↓	Endocrine modulation	Fan et al., 2019	LC-MS

HDL = high-density lipoproteins, VLDL/LDL = very-low density lipoproteins/ low density lipoproteins, FFA = free fatty acid, PC = phosphatidylcholine, PE = phosphatidylethanolamine, LPC (LysoPC) = lysophosphatidylcholine, LPE = lysophospha-tidylethanolamine, SM = sphingomyelin, DG = diglyceride, TG = trigliceride, PG = phosphatidylglycerol, AAA = aromatic amino acids, BCAA = branched-chain amino acid, AAs = amino acid, GMP = guanosine monophosphate, DHEAS = dehydro-epiandrosterone sulphate, ANDS = androsterone sulphate, DHTS = dihydrotestosterone sulphate, EPA = eicosapentaenoic acid, HODE = hydroxyoctadecadienoic acid, PEA = palmitoylethanolamide, AEA = N-arachidonoylethanolamine; ↑ up-regulation; ↓ down-regulation.

**Table 2:** The most significant changes in urinary metabolites in women with PCOS in comparison with the control subjects.

Metabolites	PCOS vs. Control	Metabolic Pathways	Studies	Techniques
Lactose	↑	Carbohydrate metabolism	Zou et al., 2018	GC-MS
Gluconic acid	↑	Carbohydrate metabolism	Zou et al., 2018	GC-MS
3-hydroxypropionic acid	↑	Carbohydrate metabolism	Zou et al., 2018	GC-MS
Arabinitol	↑	Carbohydrate metabolism	Zou et al., 2018	GC-MS
Fucose	↑	Carbohydrate metabolism	Zou et al., 2018	GC-MS
Oxalic acid	↑	Carbohydrate metabolism	Zou et al., 2018	GC-MS
Arabic candy	↑	Lipid metabolism	Zou et al., 2018	GC-MS
Stearic acid	↑	Lipid metabolism	Zou et al., 2018	GC-MS
Palmitic acid	↑	Lipid metabolism	Zou et al., 2018	GC-MS
Phosphoethanolamine	↑	Lipid metabolism	Zou et al., 2018	GC-MS
2-(14,15-Epoxyeicosatrienoyl)	↓	Glycerolipids	Wang et al., 2015	LC-MS
TG (14:1(9Z)/14:0/22:2(13Z,16Z))	↓	Glycerolipids	Wang et al., 2015	LC-MS
TG (14:0/24:1(15Z)/14:1(9Z))	↓	Glycerolipids	Wang et al., 2015	LC-MS
TG(16:0/14:0/18:0)	↓	Glycerolipids	Wang et al., 2015	LC-MS
TG (16:0/14:1(9Z)/20:1(11Z))	↓	Glycerolipids	Wang et al., 2015	LC-MS
TG	↑	Glycerolipids	Wang et al., 2015	LC-MS
DG (16:1(9Z)/14:0/0:0)	↑	Glycerolipids	Wang et al., 2015	LC-MS
PC (22:2(13Z,16Z)/18:1(9Z))	↓	Glycerophospholipids	Wang et al., 2015	LC-MS
PC (14:1(9Z)/14:1(9Z))	↓	Glycerophospholipids	Wang et al., 2015	LC-MS
LPA (16:0/0:0)	↓	Glycerophospholipids	Wang et al., 2015	LC-MS
PE (14:1(9Z)/14:1(9Z))	↑	Glycerophospholipids	Wang et al., 2015	LC-MS
LysoPC (18:1(9Z))	↑	Glycerophospholipids	Wang et al., 2015	LC-MS
Cer (d18:0/20:0)	↓	Sphingolipids	Wang et al., 2015	LC-MS
Phytosphingosine	↓	Sphingolipids	Wang et al., 2015	LC-MS
Glycocholic acid	↓	Steroids	Wang et al., 2015	LC-MS
Chenodeoxycholic acid 3-sulphate	↓	Steroids	Wang et al., 2015	LC-MS
3-Oxo-4,6-choladienoic acid	↓	Steroids	Wang et al., 2015	LC-MS
Cortolone-3-glucuronide	↑	Steroids	Wang et al., 2015	LC-MS
11α-Hydroxyprogesterone	↑	Steroids	Wang et al., 2015	LC-MS
Testosterone glucuronide	↑	Steroids	Wang et al., 2015	LC-MS
Tetrahydroaldosterone-3-glucuronide	↑	Steroids	Wang et al., 2015	LC-MS
Dehydroepiandrosterone	↑	Androgen metabolism	Dhayat et al., 2018	GC-MS
16α-OH-dehydroepiandrosterone	↑	Androgen metabolism	Dhayat et al., 2018	GC-MS
Androstenediol	↑	Androgen metabolism	Dhayat et al., 2018	GC-MS
Testosterone	↑	Androgen metabolism	Dhayat et al., 2018	GC-MS
5α-DH-testosterone	↑	Androgen metabolism	Dhayat et al., 2018	GC-MS
Androstanediol	↑	Androgen metabolism	Dhayat et al., 2018	GC-MS
Androsterone	↑	Androgen metabolism	Dhayat et al., 2018	GC-MS
11β-OH-androsterone	↑	Androgen metabolism	Dhayat et al., 2018	GC-MS
Etiocholanolone	↑	Androgen metabolism	Dhayat et al., 2018	GC-MS
Estriol	↓	Estrogen metabolism	Dhayat et al., 2018	GC-MS
Suberic acid	↑	Fatty acid metabolism	Zou et al., 2018	GC-MS
3,4,5-hydroxyvaleric acid	↑	Fatty acid metabolism	Zou et al., 2018	GC-MS
(R)-3-Hydroxy-hexadecanoic acid	↓	Fatty acid metabolism	Wang et al., 2015	LC-MS
6-Keto-decanoylcarnitine	↓	Fatty acid esters	Wang et al., 2015	LC-MS
Tiglylcarnitine	↑	Fatty acid esters	Wang et al., 2015	LC-MS
Butyrylcarnitine	↑	Fatty acid esters	Wang et al., 2015	LC-MS
4-hydroxyphenylacetic acid	↑	Tyrosine metabolism	Zou et al., 2018	GC-MS
Capryloylglycine	↓	Amino acid metabolism	Wang et al., 2015	LC-MS
*N*-(7-Isocucurbinoyl)isoleucine	↑	Amino acid metabolism	Wang et al., 2015	LC-MS
Aspartylglycosamine	↑	Amino acid metabolism	Wang et al., 2015	LC-MS
α-ketoglutarate	↑	Amino acid metabolism	Zou et al., 2018	GC-MS
Threonine	↑	Amino acid metabolism	Zou et al., 2018	GC-MS
Serine	↑	Amino acid metabolism	Zou et al., 2018	GC-MS
Glycine	↑	Amino acid metabolism	Zou et al., 2018	GC-MS
5-Oxoproline	↑	Amino acid metabolism	Zou et al., 2018	GC-MS
Benzoylglycine	↑	Amino acid metabolism	Zou et al., 2018	GC-MS
Indoleacetyl glutamine	↓	Aromatic Amino acids	Wang et al., 2015	LC-MS
Flazine methyl ether	↓	Aromatic Amino acids	Wang et al., 2015	LC-MS
Succinyladenosine	↑	Aromatic Amino acids	Wang et al., 2015	LC-MS
Thyronine	↑	Aromatic Amino acids	Wang et al., 2015	LC-MS
Gamma-glutamyl-leucine	↓	Peptides	Wang et al., 2015	LC-MS
Tryptophyl-proline	↓	Peptides	Wang et al., 2015	LC-MS
Methionyl-phenylalanine	↑	Peptides	Wang et al., 2015	LC-MS
Phenylalanyl-histidine	↑	Peptides	Wang et al., 2015	LC-MS
Arginyl-valiney	↑	Peptides	Wang et al., 2015	LC-MS
Threoninyl-lysine	↑	Peptides	Wang et al., 2015	LC-MS
Tryptophyl-arginine	↑	Peptides	Wang et al., 2015	LC-MS
Tyrosyl-leucine	↑	Peptides	Wang et al., 2015	LC-MS
Tryptophyl-valine	↑	Peptides	Wang et al., 2015	LC-MS
Cis-aconitic acid	↑	CTA metabolism	Zou et al., 2018	GC-MS
3-Hydroxy-3-Methylglutaric acid	↑	Energy metabolism	Zou et al., 2018	GC-MS
2-Hydroxyglutaric acid	↑	Energy metabolism	Zou et al., 2018	GC-MS
Threonic acid	↑	Sugar acids metabolism	Zou et al., 2018	GC-MS
Inosine	↑	Purine metabolism	Zou et al., 2018	GC-MS
2,3,4-Hydroxybutyric acid	↑	Energy metabolism	Zou et al., 2018	GC-MS
3,4-Hydroxybutyric acid	↑	Energy metabolism	Zou et al., 2018	GC-MS
4-Hydroxybutyric acid	↑	Energy metabolism	Zou et al., 2018	GC-MS
2-Hydroxyisobutyric acid	↑	Energy metabolism	Zou et al., 2018	GC-MS
Uracil	↑	Pyrimidine metabolism	Zou et al., 2018	GC-MS
Glyceryl acid	↑	Hydroxy acid metabolism	Zou et al., 2018	GC-MS
Glycolic acid	↑	Hydroxy acid metabolism	Zou et al., 2018	GC-MS
2-Hydroxyisobutyric acid	↑	Energy metabolism	Zou et al., 2018	GC-MS
Succinic acid	↓	Glucose metabolism	Zou et al., 2018	GC-MS
Benzophenone	↑	Acetophenones	Wang et al., 2015	LC-MS
5′-Carboxy-γ-chromanol	↓	Benzopyrans	Wang et al., 2015	LC-MS
5′-Carboxy-α-chromanol	↓	Benzopyrans	Wang et al., 2015	LC-MS
9′-Carboxy-α-chromanol	↓	Benzopyrans	Wang et al., 2015	LC-MS
11′-Carboxy-α-tocotrienol	↓	Benzopyrans	Wang et al., 2015	LC-MS
FMNH2	↑	Pteridines	Wang et al., 2015	LC-MS
Urobilin	↓	Tetrapyrroles	Wang et al., 2015	LC-MS
Mesobilirubinogen	↓	Tetrapyrroles	Wang et al., 2015	LC-MS
Harderoporphyrinogen	↓	Tetrapyrroles	Wang et al., 2015	LC-MS
MG (18:4(6Z,9Z,12Z,15Z)/0:0/0:0)	↑	Lineolic acids	Wang et al., 2015	LC-MS
Hydroxyvalerylcarnitine	↑	Alkylamines	Wang et al., 2015	LC-MS
Labadoside	↑	Glycosides	Wang et al., 2015	LC-MS
Dihydrocaffeic acid 3-O-glucuronide	↓	Sugar acids	Wang et al., 2015	LC-MS
Dihydroferulic acid 4-O-glucuronide	↓	Sugar acids	Wang et al., 2015	LC-MS
5-Hydroxy-6-methoxyindole glucuronide	↑	Sugar acids	Wang et al., 2015	LC-MS
p-Cresol glucuronide	↓	Sugar acids	Wang et al., 2015	LC-MS
6β-OH-cortisol	↑	Glucocorticoid metabolism	Dhayat et al., 2018	GC-MS
18-OH-cortisol	↑	Glucocorticoid metabolism	Dhayat et al., 2018	GC-MS
TH-cortisol	↑	Glucocorticoid metabolism	Dhayat et al., 2018	GC-MS
11β-OH-etiocholanolone	↑	Glucocorticoid metabolism	Dhayat et al., 2018	GC-MS
TH-cortisone	↑	Glucocorticoid metabolism	Dhayat et al., 2018	GC-MS

TG = triglyceride, DG = diglyceride, PC = phosphatidylcholine, (LysoPC) = lysophosphatidylcholine, LPA = lysophosphatidic acid, FMNH2 = reduced flavin mononucleotide; ↑ up-regulation; ↓ down-regulation.

**Table 3:** The most significant changes in FF metabolites in women with PCOS in comparison with the control subjects.

Metabolites	PCOS vs. Control	Metabolic Pathways	Studies	Techniques
Paxilline	↓	Naphthopyrans	Liu et al., 2018	LC-MS
PC (o-22:0/20:4(8Z,11Z,14Z,17Z))	↓	Glycerophospholipid	Liu et al., 2018	LC-MS
PC (o22:0/22:6(4Z,7Z,10Z,13Z,16Z,19Z))	↓	Glycerophospholipid	Liu et al., 2018	LC-MS
LysoPC (16:1(9Z))	↑	Glycerophospholipid	Liu et al., 2018	LC-MS
LysoPC (16:0)	↑	Glycerophospholipid	Liu et al., 2018	LC-MS
			Sun et al., 2019	LC-MS
LysoPC (14:0)	↑	Glycerophospholipid	Sun et al., 2019	LC-MS
LysoPC (18:0)	↑	Glycerophospholipid	Sun et al., 2019	LC-MS
LysoPC (20:4(8Z,11Z,14Z,17Z))	↑	Glycerophospholipid	Liu et al., 2018	LC-MS
PGP (16:0/20:4(5Z,8Z,11Z,14Z)	↓	Glycerophospholipid	Liu et al., 2018	LC-MS
Glycerophosphocholine	↑	Glycerophospholipid	Chen et al., 2020	LC-MS
Ceramide (d18:0/16:0)	↓	Sphingolipids	Liu et al., 2018	LC-MS
Ceramide (d18:0/24:0)	↓	Sphingolipids	Liu et al., 2018	LC-MS
Galabiosylceramide (d18:1/24:1(15Z))	↓	Sphingolipids	Liu et al., 2018	LC-MS
Tetrahexosylceramide (d18:1/24:0)	↓	Sphingolipids	Liu et al., 2018	LC-MS
7β-Hydroxycholesterol	↓	Lipid metabolism	Chen et al., 2020	LC-MS
Malyl-CoA	↓	Fatty Acyls	Liu et al., 2018	LC-MS
1-Hydroxy-2,12,15-heneicosatrien-4-one	↑	Fatty Acyls	Liu et al., 2018	LC-MS
16-hydroxypalmitic acid	↓	Fatty Acyls	Liu et al., 2018	LC-MS
Tridecanol	↓	Fatty Acyls	Liu et al., 2018	LC-MS
Carnitine	↑	Fatty acids metabolism	Chen et al., 2020	LC-MS
4-Hydroxy-3-(16-methylheptadecyl)-2H-pyran-2-one	↓	Pyrans	Liu et al., 2018	LC-MS
Anandamide	↓	Organonitrogen compounds	Liu et al., 2018	LC-MS
Indan-1-ol	↑	Indanes	Liu et al., 2018	LC-MS
2-p-Tolyl-1-propene, p-Mentha-1,3,5,8-tetraene	↑	Phenylpropenes	Liu et al., 2018	LC-MS
β -Ionol	↓	Sesquiterpenoids	Liu et al., 2018	LC-MS
Androstenol	↓	Androstane steroids	Liu et al., 2018	LC-MS
(3R, 6′Z)-3,4-Dihydro-8-hydroxy-3-(6-pentadecenyl)-1H-2-benzopyran-1-one	↓	Benzopyrans	Liu et al., 2018	LC-MS
6-Tridecylsalicylic acid	↓	Benzoic acids and derivatives	Liu et al., 2018	LC-MS
2,3-dihydroxypropyl dodecanoate	↑	Glycerol metabolism	Chen et al., 2020	LC-MS
Methylmalonic acid	↓	Carboxylic acids	Liu et al., 2018	LC-MS
Lysyl-Valine	↓	Carboxylic acids and derivatives	Liu et al., 2018	LC-MS
Prolyl-Methionine	↓	Carboxylic acids and derivative	Liu et al., 2018	LC-MS
VPGPR Enterostatin	↓	Carboxylic acids and derivative	Liu et al., 2018	LC-MS
1H-Indol-3-ylacetyl-myo-inositol	↓	Indoles and derivatives	Liu et al., 2018	LC-MS
1-Pentadecene	↓	Unsaturated hydrocarbons	Liu et al., 2018	LC-MS
Lithocholic acid glycine conjugate	↓	Non classified	Liu et al., 2018	LC-MS
Lactate	↓	Gluconeogenesis/Glycolysis	Zhang et al., 2017	NMR
	↓		Liu el al., 2018	GC-MS
Glyceraldehyde	↑	Gluconeogenesis/Glycolysis	Chen et al., 2020	LC-MS
Pyruvate	↓	Glucose glycolysis	Zhang et al., 2017	NMR
	↑		Zhao et al., 2015	GC-MS
Valine	↑	Amino acid metabolism	Zhao et al., 2015	MS/MS
Isoleucine	↑	Amino acid metabolism	Zhao et al., 2015	MS/MS
Leucine	↑	Amino acid metabolism	Zhao et al., 2015	MS/MS
			Sun et al., 2019	LC-MS
Alanine	↓	Amino acid metabolism	Zhang et al., 2017	NMR
Glutamine	↓	Amino acid metabolism	Zhang et al., 2017	NMR
Tyrosine	↓	Amino acid metabolism	Zhang et al., 2017	NMR
Phenylalanine	↑	Amino acid metabolism	Sun et al., 2019	LC-MS
d-Glutamic acid	↑	Amino acid metabolism	Chen et al., 2020	LC-MS
Ferulic acid	↑	Amino acid metabolism	Chen et al., 2020	LC-MS
Salicylic acid	↑	Amino acid metabolism	Chen et al., 2020	LC-MS
Lysine	↑	Amino acid metabolism	Chen et al., 2020	LC-MS
3-Methylhistidine	↑	Amino acid metabolism	Chen et al., 2020	LC-MS
α-Keto-β-methylvalerate	↑	Alpha-keto acids	Zhao et al., 2015	GC-MS
α-Ketoisovalerate	↑	Alpha-keto acids	Zhao et al., 2015	GC-MS
α-Ketoisocaproate	↑	Alpha-keto acids	Zhao et al., 2015	GC-MS
Hexanoyl (C6)	↓	Acylcarnitines	Zhao et al., 2015	MS/MS
Malonyl (C3DC)	↓	Acylcarnitines	Zhao et al., 2015	MS/MS
Hydroxyisovaleryl (C5OH)	↓	Acylcarnitines	Zhao et al., 2015	MS/MS
Octenoyl (C8:1)	↓	Acylcarnitines	Zhao et al., 2015	MS/MS
Adipyl (C6DC)	↓	Acylcarnitines	Zhao et al., 2015	MS/MS
β-Hydroxybutyrate	↑	Ketones	Zhao et al., 2015	GC-MS
Succinate	↑	TCA cycle metabolites	Zhao et al., 2015	GC-MS
Malate	↑	TCA cycle metabolites	Zhao et al., 2015	GC-MS
Oxaloacetate	↑	TCA cycle metabolites	Zhao et al., 2015	GC-MS
Cis-aconitate	↓	TCA cycle metabolites	Zhao et al., 2015	GC-MS
Acetate	↑	TCA cycle metabolites	Zhang et al., 2017	NMR
Acetoacetate	↑	TCA cycle metabolites	Zhang et al., 2017	NMR
3-Hyroxybutyrate	↑	TCA cycle metabolites	Zhang et al., 2017	NMR
*N*-Methylnicotinamide	↓	Metabolites of NAD catabolism	Zhao et al., 2015	LC-MS/MS
N1-Methyl-2-pyridone-5-carboxamide (2PY)	↓	Metabolites of NAD catabolism	Zhao et al., 2015	LC-MS/MS
N1-Methyl-4-pyridone-3-carboxamide (4PY)	↓	Metabolites of NAD catabolism	Zhao et al., 2015	LC-MS/MS
Deoxycorticosterone	↑	Steroid metabolism	Sun et al., 2019	LC-MS
Pregnenolone	↓	Steroid metabolism	Chen et al., 2020	LC-MS
17-Hydroxyprogesterone	↓	Steroid metabolism	Chen et al., 2020	LC-MS
3-Hydroxynonanoyl carnitine	↑	Fatty acid metabolism	Sun et al., 2019	LC-MS
Eicosapentaenoic acid	↑	Fatty acid metabolism	Sun et al., 2019	LC-MS
Phytosphingosine	↓	Sphingolipid metabolism	Sun et al., 2019	LC-MS
*N*-acetylneuraminic acid	↑	Sialic acid metabolism	Chen et al., 2020	LC-MS
Pyridoxal 5′-phosphate	↑	Vitamin B6 metabolism	Chen et al., 2020	LC-MS
Purine	↑	Purines metabolism	Chen et al., 2020	LC-MS
1,3-Dimethyluracil	↑	Purines metabolism	Chen et al., 2020	LC-MS
Oxalic acid	↑	Glyoxylic acid metabolism	Chen et al., 2020	LC-MS
Phenylglyoxylic acid	↑	Glyoxylic acid metabolism	Chen et al., 2020	LC-MS

PC = phosphatidylcholine, (LysoPC) = lysophosphatidylcholine, PGP = glycerol-3-phosphate, CoA = coenzyme A; ↑ up-regulation; ↓ down-regulation.

## Abbreviations

HA	Hyperandrogenism
AnO	Anovulatory oligomenorrhea
TV-US	Transvaginal ultrasound
PCOS	Polycystic ovary syndrome
PCOM	Polycystic ovary morphology
T2DM	Type 2 diabetes mellitus
AH	Arterial hypertension
MetS	Metabolic syndrome
NAFLD	Non-alcoholic fatty liver disease
CVD	Cardiovascular disease
IR	Insulin resistance
HMDB	Human Metabolome Database
MS	Mass spectrometry
GC-MS	Gas chromatography–mass spectrometry
LC-MS	Liquid chromatography–mass spectrometry
NMR	Nuclear magnetic resonance
FF	Follicular fluid
PPARγ	Peroxisome proliferator-activated receptor γ
PUFAs	Poly-unsaturated fatty acids
IGF-BP1	Insulin-like growth factor binding protein 1
